# Evaluation of Heat Inactivation of Human Norovirus in Freshwater Clams Using Human Intestinal Enteroids

**DOI:** 10.3390/v14051014

**Published:** 2022-05-10

**Authors:** Tsuyoshi Hayashi, Yoko Yamaoka, Atsushi Ito, Takashi Kamaishi, Ryuichi Sugiyama, Mary K. Estes, Masamichi Muramatsu, Kosuke Murakami

**Affiliations:** 1 Department of Virology II, National Institute of Infectious Diseases, Tokyo 208-0011, Japan; hayashit@niid.go.jp (T.H.); yoko.yamaoka.niid@gmail.com (Y.Y.); sugi0418@niid.go.jp (R.S.); muramatsu@niid.go.jp (M.M.); 2 Production Engineering Division, Aquaculture Research Department, Fisheries Technology Institute, Japan Fisheries Research and Education Agency, Hiroshima 722-0061, Japan; itoa@affrc.go.jp; 3 Pathology Division, Aquaculture Research Department, Fisheries Technology Institute, Japan Fisheries Research and Education Agency, Minamiise 516-0913, Japan; kamaishi@affrc.go.jp; 4 Department of Molecular Virology and Microbiology, Baylor College of Medicine, Houston, TX 77030, USA; mestes@bcm.edu; 5 Department of Medicine, Baylor College of Medicine, Houston, TX 77030, USA; 6 Department of Infectious Disease Research, Institute of Biomedical Research and Innovation, Foundation for Biomedical Research and Innovation at Kobe, Kobe 650-0047, Japan

**Keywords:** norovirus, acute gastroenteritis, intestinal enteroids, heat inactivation, freshwater clam

## Abstract

Foodborne disease attributed to the consumption of shellfish contaminated with human norovirus (HuNoV) is one of many global health concerns. Our study aimed to determine the conditions of the heat-inactivation of HuNoV in freshwater clams (*Corbicula japonica*) using a recently developed HuNoV cultivation system employing stem-cell derived human intestinal enteroids (HIEs). We first measured the internal temperature of the clam tissue in a water bath during boiling at 90 °C and found that approximately 2 min are required for the tissue to reach 90 °C. Next, GII.4 HuNoV was spiked into the center of the clam tissue, followed by boiling at 90 °C for 1, 2, 3, or 4 min. The infectivity of HuNoV in the clam tissue homogenates was evaluated using HIEs. We demonstrated that HuNoV in unboiled clam tissue homogenates replicated in HIEs, whereas infectivity was lost in all boiled samples, indicating that heat treatment at 90 °C for 1 min inactivates HuNoV in freshwater clams in our current HIE culture system. To our knowledge, this is the first study to determine the thermal tolerability of HuNoV in shellfish using HIEs, and our results could be informative for developing strategies to inactivate HuNoV in shellfish.

## 1. Introduction

Foodborne diseases attributed to the consumption of unsafe foods, which are contaminated with pathogens (bacteria, viruses, or parasites) or toxic chemical substances, cause 600 million people illness worldwide every year and therefore pose a major public health concern [1,2]. Foodborne disease is caused by several pathogens, including *Campylobacter*, *Salmonella*, *Listeria*, hepatitis A virus, and human norovirus (HuNoV). Among those, HuNoV is the most frequently detected pathogen in contaminated foods being consumed by ill individuals [1,3]. Shellfish, especially oysters, are recognized as one of the major sources for HuNoV-associated foodborne disease due to the accumulation of HuNoVs in the digestive gland by filter feeding [4].

The most common inactivation method of pathogens in contaminated foods is to cook them properly at a high temperature. Given the varied thermal tolerability of each pathogen, the establishment of tailor-made strategies to inactivate their respective pathogens is necessary to reduce the risk of foodborne illness. Concerning HuNoV, there was no robust HuNoV cultivation system developed until recently. Therefore, investigations on the food inactivation of HuNoV have been carried out by measuring copy numbers of the HuNoV genome or infectious virus titers of surrogate viruses, such as murine norovirus (MNV) and feline calicivirus (FCV) [5,6]. However, it remains unclear whether these indirect measures reflect the inactivation of HuNoV infectivity.

Recently, several HuNoV cultivation systems have been developed, including B cells [7], tissue stem cell-derived human intestinal enteroids (HIEs) [8,9], human induced pluripotent stem cell-derived intestinal epithelial cells (iPSC-derived IECs) [10] and zebrafish [11]. Studies using our HIE system, as well as human iPSC-derived IECs, show HuNoV inactivation by heating or disinfectants (alcohol or chlorine) [8,12,13]. However, to our knowledge, an investigation on HuNoV’s inactivation in foods such as bivalves using the HuNoV cultivation system has not been performed so far.

In this study, we evaluated the thermal inactivation conditions of freshwater clams artificially inoculated with HuNoV by measuring infectious HuNoV using the HIE culture system.

## 2. Materials and Methods

### 2.1. Measurement of Temperature Kinetics in Freshwater Clams Subjected to Heat Treatment

**Figure 1:**
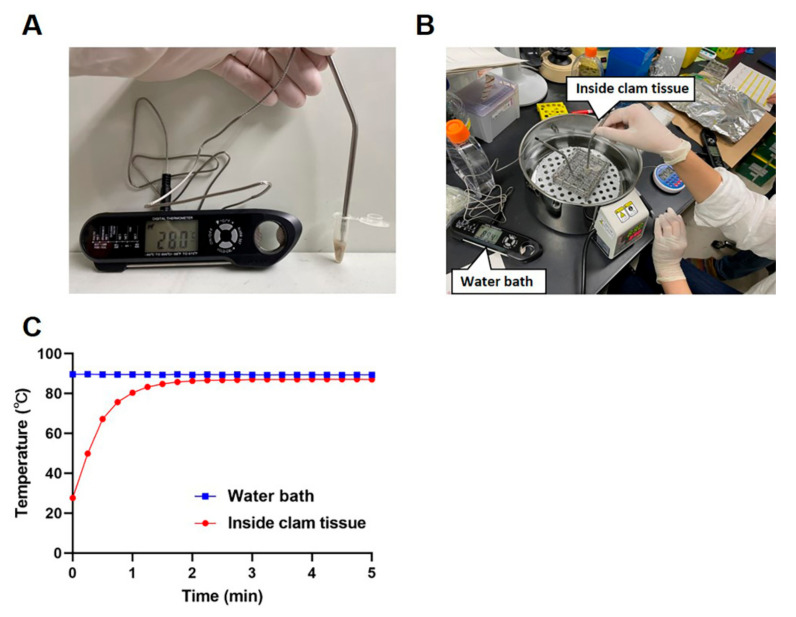
Kinetics of the internal temperature in clams subjected to heat treatment. A thermometer probe was inserted into a clam body in a 1.5-mL tube to measure the internal temperature of the clam (**A**). A second thermometer probe was put in the water bath to measure the temperature outside the 1.5-mL tube (**B**). The internal clam tissue temperature and the external water bath temperature were recorded every 15 s up to 5 min (**C**). The results are shown as the mean ± standard deviation calculated from 5 independent experiments (*n* = 5).

Live freshwater clams (*Corbicula japonica*) were purchased at a grocery store and maintained overnight at room temperature in diluted seawater. A whole clam body was then taken from its shell and transferred into a 1.5-mL tube. The weights of the clam bodies ranged from 0.16 g to 0.41 g (mean ± standard deviation (SD), 0.29 ± 0.07). To monitor the internal and external temperatures of the samples, two thermometers were used; a probe thermometer was inserted into a clam body (Figure 1A), and another was immersed in a water bath set at 90 °C (Figure 1B). Both temperatures were recorded every 15 s up to 5 min.

### 2.2. Preparation of HuNoV-Containing Stool Filtrate

Ten percent stool filtrate containing HuNoV was prepared as described previously with minor modifications [8]. Briefly, PBS was added to a HuNoV-positive stool and homogenized by vortexing and sonication. The suspensions were centrifuged at 12,000× *g* for 5 min at 4 °C, and the supernatant was passed serially through 5-µm and 0.22-µm filters. The samples were aliquoted and frozen at −80 °C before use.

### 2.3. Artificial Inoculation of HuNoV into Freshwater Clams Followed by Heat Treatment and Sample Processing

Six freshwater clams (one clam per each time point plus PBS control) were used in one experiment, and the experiment was repeated three times. Each clam body taken from its shell was injected with 30 μL of either PBS as a control or 10% stool filtrate containing 1.06 × 10^8^ genome equivalents (GEs) of GII.4[GII.P16] HuNoV [14] using a 50-μL microsyringe with a fine needle. The relatively high titer of HuNoV was used for this purpose, because the resultant clam homogenate needed to be diluted to minimize its cytotoxicity in HIEs. The artificial inoculated samples were then left untreated or heat-treated at 90 °C for 1, 2, 3, or 4 min, as described above. After cooling the samples down to room temperature, 170 μL of chilled complete medium without growth factors (CMGF(-)) used for culturing HIEs was added to each sample. The clam bodies were then chopped with scissors and homogenized using a hand mixer (PowerMasher II, 891300, nippi, Tokyo, Japan) for 30 s, followed by centrifugation at 9100× *g* for 3 min at 4 °C. The supernatant was collected and repeated the centrifugation with the same condition to remove debris. The collected supernatant was stored at −80 °C until used for determining the virus infectivity and viral recovery efficiency. The final recovered volume was 160 ± 26 μL (PBS, 0 min), 193 ± 25 μL (HuNoV, 0 min), 210 ± 17 μL (HuNoV, 1 min), 177 ± 45 μL (HuNoV, 2 min), 220 ± 26 μL (HuNoV, 3 min), or 223 ± 23 μL (HuNoV, 4 min).

### 2.4. Evaluation of Infectivity of HuNoV in Clam Extracts Using HIEs

HIE culture and HuNoV infection were performed as described previously [8,14,15]. Briefly, a jejunal HIE (J2) culture, provided from the Baylor College of Medicine under the Material Transfer Agreement, was maintained and propagated as Matrigel-embedded, 3-dimensional (3D) HIEs in complete medium with growth factors (CMGF(+)) or IntestiCult Organoid Growth Medium (Human, STEMCELL, Vancouver, BC, Canada).

To prepare monolayer HIE cultures for HuNoV infection, the 3D HIEs (passages 25–28) were dissociated with TrypLE Express (Thermo Fisher, Waltham, MA USA) and seeded onto collagen IV-coated 96-well plates at the number of approximately ~10^5^ cells/well in the CMGF(+) or IntestiCult media supplemented with ROCK inhibitor Y-27632 (10 μM, Sigma, Burlington, MA, USA) for 2 days. The medium was then replaced with IntestiCult Organoid Differentiation Medium (Human, STEMCELL), and the cultures were maintained for an additional 2 days. The monolayer HIEs were then inoculated with 5 μL of the clam samples diluted 1:20 in a final volume of 100 μL of CMGF(-) medium in the presence of 500 μM GCDCA, which promotes GII.4 HuNoV infection [8]. After 1 h of incubation at 37 °C, the cells were washed twice with CMGF(-) and further incubated in IntestiCult Organoid Differentiation Medium containing 500 μM GCDCA until 24 h post-infection (hpi). The cells and medium were then collected and subjected to RNA extraction using the Direct-zol RNA MiniPrep kit (Zymo Research, Irvine, CA, USA) following the manufacturer’s instructions. The RNA was eluted in 50 μL of distilled water.

HuNoV RNA genome equivalents (GEs) were determined by reverse transcription-quantitative PCR (RT-qPCR) analysis in a 20 μL reaction volume using a TaqMan Fast Virus 1-Step Master Mix (Thermo Fisher) and GII specific primer/probe sets [16]. Five microliters of RNA in each sample were used for this assay. A standard curve generated using plasmid-containing HuNoV genome sequences was used to quantitate viral GEs. We defined the limit of detection in the RT-qPCR analysis as 20 GEs/rxn (3.0 log_10_ GEs/well) based on a standard curve.

### 2.5. Evaluation of Viral Recovery Efficiency after Homogenization

The RNA was extracted from 5 μL of the inoculum containing 30 μL of HuNoV-containing stool filtrates mixed with 170 μL of CMGF(-) (input) or the HuNoV-inoculated clam extracts (samples) and then subjected to RT-qPCR analysis to measure the HuNoV GEs. The percentage of recovery efficiency was calculated as HuNoV GEs in clam samples relative to that in the inoculum.

### 2.6. Statistical Analysis

Statistical analysis was performed with ANOVA, followed by Dunnett’s multiple-comparison test or two-tailed Student’s *t*-test using GraphPad Prism 9 software. *p*-values of <0.05 were considered statistically significant.

## 3. Results

Heat treatment experiments were carried out using a water bath at 90 °C. Measurements of the internal and external temperatures during heating showed that, while the water temperature was stable at around 90 °C during the heating process, the temperature of the clam body required 2 min before reaching a stable temperature at 90 °C (Figure 1C).

**Figure 2:**
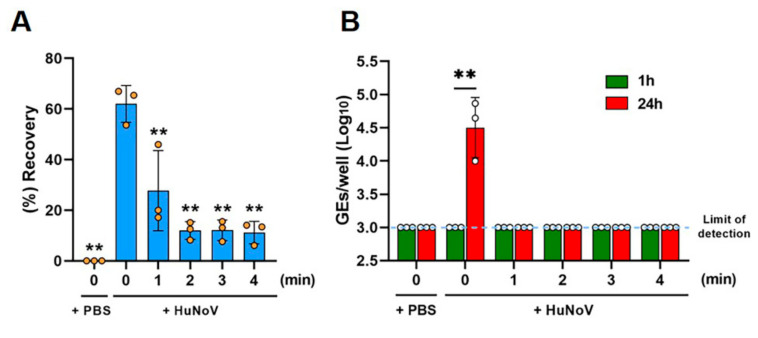
Thermal inactivation of HuNoV in freshwater clams. The clam tissue was spiked with either PBS or HuNoV-containing stool filtrate. The spiked clam tissue was next left untreated or heat-treated at 90 °C for 1, 2, 3, or 4 min and then homogenized. (**A**) Viral GEs in the clam extracts were quantified by RT-qPCR, and the recovery efficiency was calculated as in the Materials and Methods. ** *p* < 0.01 vs. unheated HuNoV-spiked clam samples (0 min), one-way ANOVA followed by Dunnett’s multiple comparison test. (**B**) The samples were inoculated against differentiated HIEs, and HuNoV GEs in the HIEs were determined as in the Materials and Methods. The limit of detection in the RT-qPCR analysis is 3.0 log_10_ GEs/well. ** *p* < 0.01, two-tailed Student’s *t*-test. Results are shown as the mean ± standard the deviation calculated from 3 independent experiments (*n* = 3).

We then evaluated the effects of different times of heat inactivation on the HuNoV-inoculated clams. The clam bodies were either spiked with PBS as a non-infection control or HuNoV using a microsyringe. The inoculated clam tissue, either left unheated or heated at 90 °C for 1, 2, 3, or 4 min, was homogenized. We first evaluated the recovery efficiency of spiked HuNoV in clam homogenates (Figure 2A and Figure S1). Approximately 60% of the inoculated HuNoV GEs (62.0 ± 7.3%) were recovered from HuNoV-spiked clam homogenates without heating, whereas the treatment at 90 °C for 1 min significantly reduced the recovery (27.7 ± 15.9%) as compared to the unheated samples. The treatment at 90 °C for 2 min further reduced the recovery (12.0 ± 3.5%), while the recovery remained unchanged thereafter (Figure 2A). In addition, to see if heated clam homogenates affect the recovery of HuNoV, we quantified HuNoV RNA extracted from pre- or post-heated clams inoculated with HuNoV. The results showed no significant difference of viral GEs between them (Figure S2).

Then, the infectious virus was quantified, following replication in the culture, by determining the viral GEs at 1 or 24 hpi, as described in the Materials and Methods section. HuNoV in clam homogenates was infectious and able to be replicated in HIEs showing a 42.5-fold increase in GEs at 24 hpi (4.5 ± 0.5 GEs/well) as compared to 1 hpi (3.0 ± 0.0 GEs/well), while the PBS-injected samples contained no infectious virus, as expected (Figure 2B). Furthermore, all groups of heat treatment showed no viral replication at 24 hpi, suggesting that the 90 °C for 1 min treatment inactivates HuNoV in clam bodies (Figure 2B), although improvement of the viral growth efficiency in HIEs is required for optimal inactivation studies, as discussed below.

## 4. Discussion

Generally, if no cultivation system is available to grow a certain human pathogen, employing cultivable surrogate virus(es) is used to study the biological characteristics, including tolerability against disinfectants or heating [6]. However, the properties of the surrogate viruses are not always the same as the human pathogen. Indeed, previous reports demonstrated that HuNoV is resistant to 70% alcohol [12], whereas surrogate viruses such as murine norovirus (MNV), feline calicivirus (FCV) and porcine enteric calicivirus, but not Tulane virus, are sensitive to this treatment [17]. A study on heat inactivation of MNV in shellfish showed that the heat treatment at 90 °C for 90 s resulted in an approximate 2 log_10_ reduction of the infectious virus measured by using plaque assays [5]. The authors also demonstrated that the 90 °C for 180 s completely inactivated MNV and hepatitis A virus (HAV), another important human pathogen related to viral foodborne illness [5]. Whether the heat treatment at 90 °C for 1 min is sufficient for the inactivation of MNV or HAV, as well as HuNoV, remains to be verified.

Recently, a propidium monoazide (PMA) viability RT-qPCR assay, which is expected to measure only infectious virions containing intact viral RNA but not non-infectious virions containing degraded RNA, was applied to study the inactivation of HuNoV in clams [18]. That study demonstrated that HuNoV spiked in clams and heated at 90 °C for 10 min resulted in a 3.52 log_10_ reduction of HuNoVs determined by a PMA viability RT-qPCR assay [18]. Since the growth of HuNoVs in HIEs is still lower than that of surrogate viruses in their cultivable cells, improvement of the growth efficiency is required to be utilized as general evaluation methods for HuNoV inactivation. For example, conditions to cultivate HuNoV in genetically modified lines and with improved medium conditions to achieve at least three logs of replication are being sought for optimal inactivation studies [9,19]. In addition, the improvement of the HuNoV quantification method (e.g., lowering of the limit of detection in RT-qPCR analysis) is required for applying to actual contaminated samples that might be destined for heat inactivation. Meanwhile, comparisons between the PMA viability RT-qPCR assay and our evaluation method using HIEs would be beneficial to develop a general pragmatic method to evaluate HuNoV inactivation.

In this study, we used freshwater clams to evaluate HuNoV inactivation using HIEs, because the HuNoV genome is frequently detected in clams, as well as oysters, among bivalve mollusks [20]. It would be worth comparing HuNoV inactivation patterns in between clams and oysters, which needs further investigation.

In summary, we evaluated the heat inactivation of HuNoV in freshwater clams using HIEs and showed that treatment at 90 °C for 1 min inactivates HuNoV in the clam bodies. Although our current HIE system is suboptimal for evaluating actual contaminated samples, this information could be valuable when developing guidelines to inactivate HuNoV, which will contribute to reducing the risk of foodborne illness associated with HuNoV.

## Data Availability

All data are presented in the article, and further inquiries can be directed to the corresponding author.

## Supplementary Materials

The following supporting information can be downloaded at https://www.mdpi.com/article/10.3390/v14051014/s1: Figure S1: Raw data for Figure 2A. Recovery of HuNoV in freshwater clams after heat treatment. Figure S2: Recovery of HuNoV in the presence of heated clam tissue.
