# Supplementary material for: Evaluation of Heat Inactivation of Human Norovirus in Freshwater Clams Using Human Intestinal Enteroids
Source: Viruses. 2022 May 10;14(5):1014. doi: 10.3390/v14051014 (PMC9146323; doi:10.3390/v14051014)
Supplement: Supplementary file 1 [file viruses-14-01014-s001.zip › viruses-1669920-supplementary.pdf]

viruses

MDPI

Supplementary Material

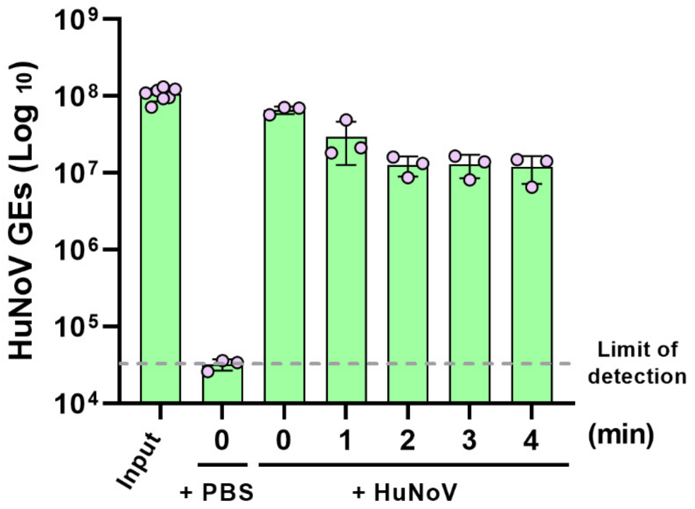

Figure S1. Raw data for Figure 2A; Recovery of HuNoV in freshwater clams after heat treatment.

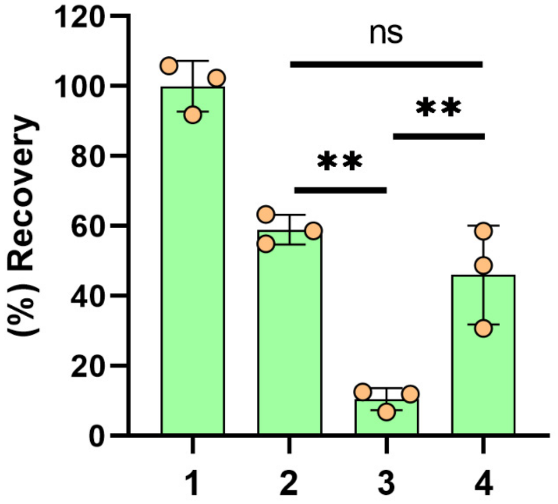

Figure S2. Recovery of HuNoV in the presence of heated clam tissue. Viral GEs in pre- or post-heated clam extracts were quantified by RT-qPCR and the recovery was calculated as in Materials and methods. ** $p < 0.01$, one-way ANOVA followed by Tukey's multiple-comparison test. ns; not significant ($p > 0.05$). 1. Input; 2. Clam inoculated with HuNoV without heat treatment (0 min); 3. Clam inoculated with HuNoV followed by heat treatment (90 °C, 4 min); 4. Pre-heated (90°C, 4 min) clam inoculated with HuNoV.
